# Asperaculanes A and B, Two Sesquiterpenoids from the Fungus *Aspergillus aculeatus*

**DOI:** 10.3390/molecules20010325

**Published:** 2014-12-25

**Authors:** Yu-Qi Gao, Chun-Jun Guo, Qiang Zhang, Wen-Ming Zhou, Clay C. C. Wang, Jin-Ming Gao

**Affiliations:** 1 Shaanxi Key Laboratory of Natural Products Chemical Biology, College of Science, Northwest A&F University, Yangling 712100, Shaanxi, China; E-Mails: gaoyuqi1989@gmail.com (Y.-Q.G.); zerkang@126.com (Q.Z.); zhouwenming2008@nwsuaf.edu.cn (W.-M.Z.); 2 Department of Pharmacology and Pharmaceutical Sciences, School of Pharmacy, University of Southern California, Los Angeles, CA 90089, USA; E-Mail: chunjung@usc.edu

**Keywords:** *Aspergillus aculeatus*, nordaucane sesquiterpenoids, *ent*-daucanes, natural products, structure elucidation

## Abstract

Six sesquiterpenoids **1**–**6**, including two new ones, an *ent*-daucane-type sesquiterpenoid, asperaculane A (**1**), and a nordaucane one, asperaculane B (**2**), and four known nordaucane derivatives, aculenes A–D **3**–**6**, together with the known secalonic acid D (**7**), were isolated from a fermentation culture of the fungus *Aspergillus aculeatus*. Their structures and absolute configurations were established by analyses of their spectroscopic data, including 1D and 2D-NMR spectra, HR-ESIMS, electronic circular dichroism (ECD) data, and quantum chemical calculations. These metabolites were evaluated for *in vitro* cytotoxic activity against two cell lines, human cancer cell lines (HeLa) and one normal hamster cell line (CHO).

## 1. Introduction

Fungi are abundant sources of secondary metabolites with distinct structures and a broad range of intriguing biological activities [1,2,3,4]. The genus *Aspergillus* (Moniliaceae), with over 180 species, has attracted considerable attention as a rich source of alkaloids, terpenoids, xanthones, steroids, and polyketides, some of which showed antimicrobial, antifouling, antifeedant, phytotoxic, or other interesting bioactivities [5,6,7,8,9,10]. For example, *Aspergillus aculeatus* has been found to generate a variety of bioactive natural products, such as aculeacins A–G (antibiotics and antifungal agents) [11,12], CJ-15,183 (squalene synthase inhibitor and antifungal agent) [13], aspergillusol A (α-glucosidase inhibitor) [14], secalonic acids D and F (toxins) [15], asperparaline A [16], cytotoxic aculeatusquinones A–D [17], and two okaramine alkloids okaramines H and I [18].

In order to identify new bioactive natural compounds from various *Aspergillus* fungi, we studied the chemical constituents of the cultures of *A. aculeatus*. Herein we report the isolation and structure elucidation of two new sesquiterpenoids, named asperaculanes A (**1**) and B (**2**).

## 2. Results and Discussion

**Figure 1:**
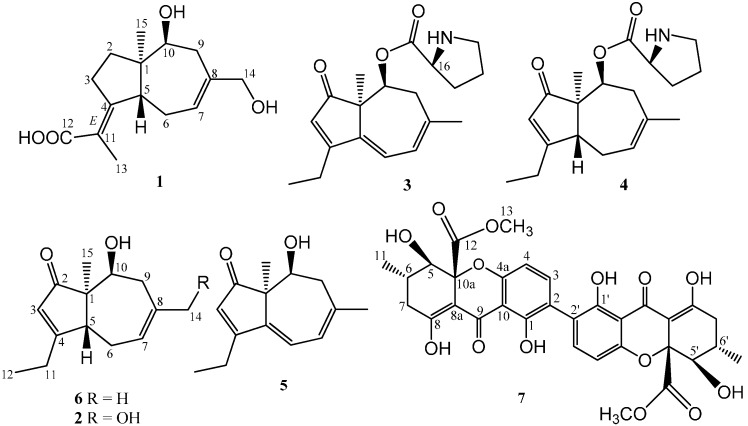
Chemical structures of compounds **1**–**7** from *A. aculeatus*.

The CH_2_Cl_2_/MeOH (1:1) extract of solid cultures of *A. aculeatus* was evaporated, and the resulting residue was extracted with EtOAc. Purification of the EtOAc extracts by silicagel column chromatography and reversed-phase preparative HPLC afforded two new sesquiterpenens **1** and **2**, along with the known metabolites **3**−**7** (Figure 1).

### 2.1. Structure Elucidation of Compounds **1**–**2** and Absolute Configurations of **3**–**6**

Asperaculane A (**1**) has the molecular formula C_15_H_22_O_4_, with five degrees of unsaturation, as established by a quasimolecular ion peak at *m/z* 289.1418 [M+Na]^+^ (calcd. 289.1416) in HR-ESIMS. The UV spectrum shows absorption for α,β-unsaturated carboxylic acid or ketone at 241 nm. The IR spectrum has characteristic bands of hydroxyls and a conjugated carboxylic acid at 3345 and 1686 cm^−1^, respectively. The presence of the two hydroxyl groups in **1** was supported by HR-ESIMS fragments at *m/z* 249.1490 [M+H-H_2_O]^+^ and 231.1385 [M+H-2H_2_O]^+^.

**Figure 2:**
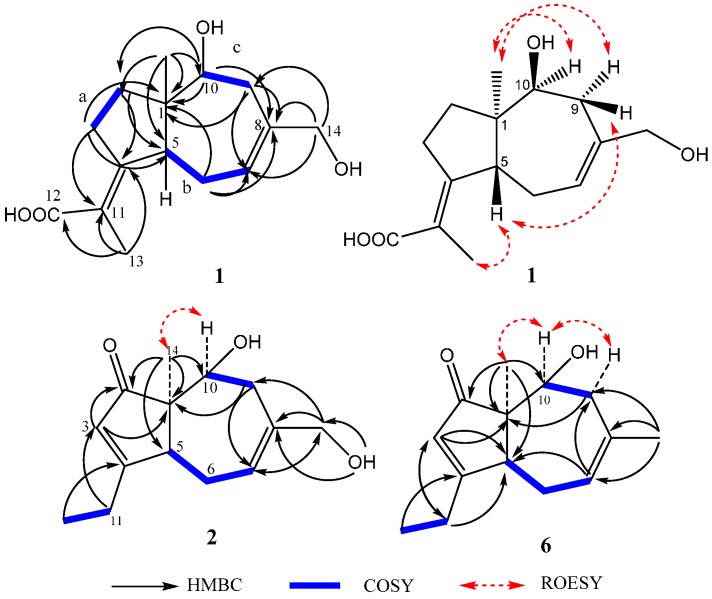
Key HMBC, COSY, and NOESY correlations of **1**, **2**, and **6**.

The ^13^C-NMR and DEPT spectra of **1** (Table 1) exhibited 15 signals due to two methyls, five sp^3^-hybridized methylenes (including one oxygenated), two sp^3^-methines (including one oxygenated), one sp^2^-methine (C-7, δ_C_ 121.3), one quaternary sp^3^-carbon (C-1, δ_C_ 48.2), and four quaternary sp^2^-carbon. The ^1^H-NMR spectrum of **1** (Table 1) displayed signals from two isolated methyls including an allylic one (δ_H_ 1.81, s, Me-13), two methines (δ_H_ 3.04, d, *J* = 11.7 Hz, H-5; 3.82, br d, *J* = 2.3 Hz, H-10), a hydroxymethylene (δ_H_ 3.69, s), and an olefinic proton (δ_H_ 5.65, d, *J* = 5.9 Hz, H-7). The presence of the carbonyl group and two double bonds accounted for three of five degrees of unsaturation, indicating that **1** possesses two rings in the molecule. Analysis of the ^1^H-^1^H-COSY spectrum for **1** revealed the connectivity of three substructures; a (C-2 to C-3), b (C-5 to C-7), and c (C-9 to C-10), as shown in Figure 2. The HMBC correlations of H-2 with C-4/C-5, H-3 with C-1/C-5/C-11, H-13 with C-4/C-11/C-12, H-6 and H-9 with C-1/C-7/C-8, H-10 with C-1/C-2/C-8, H-14 with C-7/C-8/C-9, and H-15 with C-1/C-2/C-5/C-10, allowed us to define the planar structure of **1** with a daucane or carotane skeleton.

**Table 1:** ^1^H-NMR and ^13^C-NMR spectroscopic data for compounds **1**, **6**, and **2**.

No.	1 *^a^*		6 *^b^*		2 *^a^*	
	δ_H_ (*J* in Hz)	δ_C_	δ_H_ (*J* in Hz)	δ_C_	δ_H_ (*J* in Hz)	δ_C_
1	—	50.1	—	56.8	—	56.5
2	1.20, m 1,85 *^c^*	36.7	—	213.3	—	211.0
3	2.52 *^c^* 2.72, dd (17.6, 7.0)	34.0	5.79, s	123.5	5.70, s	124.0
4	—	162.7	—	186.2	—	184.1
5	3.04, d (11.7)	44.8	3.52, d (12.6)	44.3	3.40, d (14.4)	44.0
6	2.91, m 2.52 *^c^*	28.9	2.11, d (15.2) 2.53 *^c^*	25.5	2.03, d (14.8) 2.52 *^c^*	25.5
7	5.65, d (5.9)	126.0	5.56, br d (5.7)	122.1	5.70 overlapped	121.3
8	—	137.8	—	132.4	—	137.4
9	2.34, d (17.0) 2.15, dd (17.0, 2.3)	36.1	2.32, d (18.4) 2.55 *^c^*	39.5	2.15, d (18.0) 2.32, ddd (18.4, 10.8, 4.4)	36.5
10	3.82 br. d (2.3)	73.8	4.15, dd (2.6, 4.9)	68.8	4.00, d (2.4)	68.0
11	—	122.7	2.4, q (7.3)	23.8	2.35, q (7.3)	24.0
12	—	173.2	1.19, t (7.3)	10.3	1.08, t (7.3)	11.7
13	1.81, s	16.7	—	—	—	—
14	3.69 br. s	71.1	1.74, s	28.3	3.67, d (4.8)	68.9
15	0.88, s	19.6	0.97, s	17.3	0.80, s	18.3

*^a,b^:* Data were recorded in DMSO-*d*_6_, and MeOH-*d*_4_, respectively; *^c^:* Signals were overlapped with each other or by solvents.

The stereochemistry of **1** was deduced by a NOESY experiment in MeOH-*d*_4_. The NOE correlations from H-10α (δ_H_ 3.82) and H-9α (δ_H_ 2.34) to Me-15 (δ_C_ 0.88), indicates that the Me-15 group was in the α-position, thereby implying the 10-OH is β-oriented, while the NOESY signal between H-9β (δ_H_ 2.15) and H-5 (δ_H_ 3.04), together with the absence of a NOE correlation between Me-15 and H-5, established H-5 as β. These data indicated that the bicyclic ring in **1** were *trans*-fused, as illustrated in Figure 1, providing more pieces of evidence that this compound belongs to the *ent*-daucane-type sesquiterpene. Additionally, the geometry for the ∆^4(11)^ olefin in **1** was assigned *E* on the basis of a marked NOESY cross peak between H-13 and H-5.

**Figure 3:**
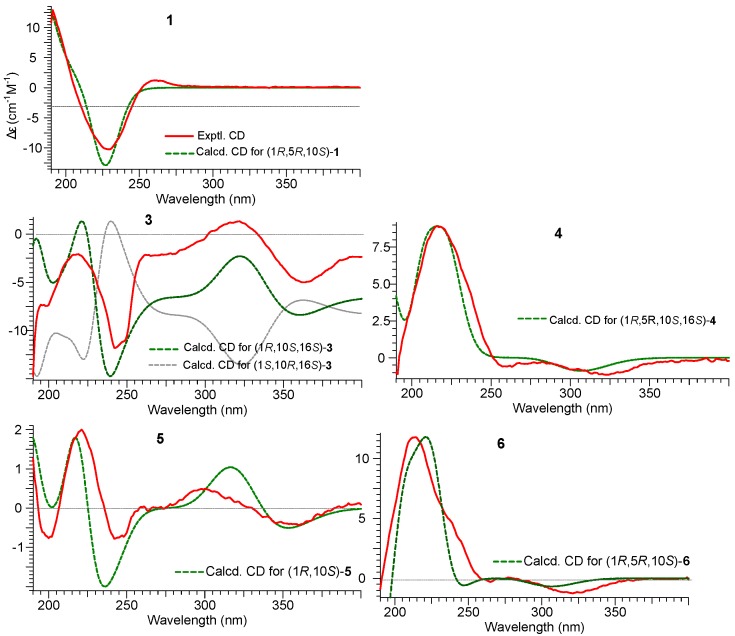
Experimental CD spectra of **1**, **3**–**6** in MeOH and theoretical CD spectraof **1**, **3**, **5**, and **6** calculated at CAM-B3LYP/TZVP//B3LYP/6-311+G(d,p) level, and **4** calculated at CAM-B3LYP/SVP//B3LYP/6-31+G(d,p) level as Boltzmann average.

Quantum chemical calculations of electronic circular dichroism (ECD) spectra have been proven to be reliable tools in determining the absolute configurations of organic molecules [19]. The CD spectrum of **1** showed two Cotton effects, including a negative first Cotton effect at 229 nm (–13.3) and the positive Cotton effect at 261 nm (∆ɛ = +1.56). To determine the absolute configuration of **1**, a comparison was made between the experimental and calculated CD spectra by the time-dependent density functional theory (TDDFT) method. Conformational searches were performed by Conflex6.7 with the MMFF94S force field [20]. Conformers within 3 kcal/mol were saved and further optimized at B3LYP/6-311+G(d,p) level in Gaussian 09 software package [21]. The stable conformers with populations greater than 1% and without imaginary frequencies were submitted to ECD calculation by the TDDFT [CAM-B3LYP/TZVP] method associated with CPCM solvent model in methanol. As a result, the calculated CD spectrum for **1** agreed well with that experimental one for **1** (Figure 3), and the absolute configuration of **1** was assigned as (1*R*,5*R*,10*S*). Thus, the structure of **1** was elucidated as 4*E*(11)-(1*R*,5*R*,10*S*)-10,14-dihydroxydauca-4(11),7-dien-13-carboxylic acid.

Compounds **3**–**5**, and **7** were characterized as aculenes A, B, C, and secalonic acid D, respectively, based on HR-ESIMS, NMR data, Marfey’s method [22] and comparison of their spectroscopic data with those reported in the literature [15,23]. Aculene D (**6**) had the molecular formula C_14_H_20_O_2_, as deduced from HR-ESIMS data (*m/z* 243.1362 [M+Na]^+^). The ^1^H- and ^13^C-NMR spectroscopic data of **6** (Table 1) had the almost similar signals as **4**, except for the absence of the signals of the proline moiety. Its structure was confirmed by analysis of the COSY, HMQC, and HMBC experiments (Figure 2). The NOESY cross-peaks between H-10α and Me-14α indicated that the OH group at C-10 was β-oriented. This compound had been detected previously in the same organism by LC-HRMS [24], but no NMR data were reported.

The molecular formula C_14_H_20_O_3_ of **2** was determined from HR-ESIMS. The NMR data of **2** (Table 1) were very similar to that of **6**. The major difference was that the methyl group at C-13 (H-13, δ_H_ 1.74, s; δ_C_ 28.3) in **6** was replaced by a hydroxymethylene one at C-13 (H-13, δ_H_ 3.67, d, *J* = 4.8 Hz; δ_C_ 68.9) in **2**. This deduction was supported by key HMBC correlations of H-13 (δ_H_ 3.67) to C-7 (δ_C_ 121.3), C-8 (δ_C_ 137.4), and C-9 (δ_C_ 36.5); 13-OH (δ_H_ 4.73) to C-13 (δ_C_ 68.9), C-8. The structure of **2** was confirmed by 2D NMR analysis (Figure 2). Biogenetically, the absolute configuration of **2** was concluded to be (-)-(1*R*,5*R*,10*S*)-**2**, the same sterechemistry as **1**. The structure of **2** was thus determined as (1*R*,5*R*,10*S*)-asperaculane B.

However, the absolute stereochemistries of **3**–**6** were unknown. To determine the absolute configuration of these compounds, we employed the experimental and calculated CD spectra. A comparison was made among the experimental and calculated CD spectra of (1*R*,10*S*,16*S*)-**3** and (1*S*,10*R*,16*S*)-**3**. The calculated CD spectrum for **3** agreed well with its experimental one (Figure 3). Thus, the absolute configuration of other chiral centers in **3** was assigned as 1*R* and 10*S*, and the structure of **3** was elucidated as (1*R*,10*S*,16*S*)-aculene A. Similarly, the absolute configuration of **5** was assigned as *(1R,10S)* from its well matched calculated and experimental CD spectra (Figure 3). Thus, the structure of **5** was established as (1*R*,10*S*)-aculene C, while the absolute configuration of **6** was assigned as *(1R*,*5R*,*10S)* from its well matched calculated and experimental CD spectra (Figure 3). Thus, the structure of **6** was established as *(1R*,*5R*,*10S)*-aculene D.

Furthermore, theoretical CD spectra of **4** were calculated at CAM-B3LYP/SVP//B3LYP/6-31+G(d,p) level. The absolute configurations of chiral centers at C-1, C-5, C-10, and C-16 in **4** were established as 1*R*, 5*R*, 10*S*, and 16*S* from its well matched calculated and experimental CD spectra (Figure 3). Thus, the structure of **4** was determined as (1*R*,5*R*,10*S*,16*S*)-aculene B.

The metabolites **1**–**6** were possibly biosynthesized from daucenes, [25] which could be sesquiterpenoid natural products of plants and fungi [24,25]. Compound **1** was proposed to be the precusor of the co-existing metabolites **2**–**6**, which was formed via a series of decarboxylation, oxidations, and dehydrogenation of **1**.

### 2.2. Cytotoxicity Assay

The isolated compounds **1**–**6** were all tested for their *in vitro* cytotoxic activities against one human cancer cell lines HeLa and one normal hamster cell line (CHO) using the SRB colorimetric assay [26]. None of the tested compounds showed any cytotoxicity below IC_50_ values of 50 μM.

## 3. Experimental Section

### 3.1. General Procedures

Optical rotations were measured on an Autopol III automatic polarimeter. ^1^H- and ^13^C-NMR spectra were recorded on a Varian Mercury Plus 400 spectrometer or a Bruker Avance III 500 spectrometer. Chemical shifts are reported in ppm relative to DMSO-*d*_6_ (^1^H, δ 2.50; ^13^C, δ 40.08) or MeOH-*d*_4_ (^1^H, δ 3.30; ^13^C, δ 49.1). ^1^H-NMR data is reported as: chemical shift, multiplicity (s = singlet, d = doublet, t = triplet, q = quartet, m = multiplet), coupling constant, and integration. Infrared (IR) spectra were run on a Bruker Tensor spectrophotometer. High resolution electrospray ionization mass (HR ESIMS) was obtained on an Agilent 6210 time of flight LC-MS. Reactions were monitored by analytical thin-layer chromatography on EMD silica gel-60F_254_ plates. Flash chromatography was performed on EMD silica gel 60, 70–230 mesh. All reagents were used without further purification unless otherwise noted.

### 3.2. Fungal Material and Fermentation

The fungal strain *Aspergillus aculeatus* ATCC16872 v1.1 was provided by the U.S. Department of Energy Joint Genome Institute (DOE JGI). The fungus was cultivated at 30 °C on 40 solid YES plates at 2.25 × 10^6^ spores per 15-cm plates (~0.1 mL of medium per plate). After 5 days, agar was cut into small pieces and put in two 2-liter flasks.

### 3.3. Extraction, Isolation and Purification

The solid agar was chopped into pieces and saturated twice in around 750 mL of 1:1 CH_2_Cl_2_/MeOH for 24 h. After filtration, the combined extract was evaporated *in vacuo* to obtain a residue, which was suspended in water (400–500 mL) and then partitioned with EtOAc three times. The combined EtOAc layer was evaporated *in vacuo* to yield a crude extract. The crude extract was applied to a silica gel column (Merck, 230 to 400 mesh, ASTM, 20 × 80 mm) and eluted with 400 mL CH_2_Cl_2_/MeOH mixtures of increasing polarity (fraction A, 1:0; fraction B, 19:1; fraction C, 9:1; fraction D, 7:3). The LC-MS profiles of these fractions suggests that fraction C (947.7 mg) had most of the compouds.

Fraction C was further purified by reverse phase HPLC [Phenomenex Luna 5 μm C18 (2), 250 × 10 mm] with a flow rate of 5.0 mL/min and measured by a UV detector at 254 nm. The gradient system was MeCN (solvent B) in 5% MeCN/H_2_O (solvent A) both containing 0.05% TFA. The HPLC gradient condition is 0% to 40% B from 0 to 20 min, 40% to 100% B from 20 to 40 min, maintained at 100% B from 40 to 42 min, 100% to 0% B from 42 to 44 min.

Fraction 3 yielded 14 subfractions (3.1–3.14), among which six subfractions (3.2, 3.3, 3.6, 3.8, 3.10, 3.14) were further purified by semi preparative HPLC. Fractions 3.2, 3.10 and 3.14 afforded compounds **1** (15.7 mg), **6** (25.9 mg) and **7** (6.6 mg), respectively. Compounds **3** (32.6 mg), **4** (57.7 mg), **2** (3.3 mg) and **5** (6 mg) were obtained from subfractions 3.3, 3.6, and 3.8, respectively.

*Asperaculane A* (**1**). Yellowish-brown oily liquid; ${\left[\text{α}\right]}_{D}^{\text{12}}$ = +27.0 (c 0.13, MeOH); UVλ_max_ = 241 nm; CD (MeOH) λ_max_ (Δε): 229 (−13.3), 261 (+1.56); IR (KBr) ν 3416, 2949, 2874, 1678, 1452, 1381, 1276, 1202, 1109, 1058, 1026 cm^−1^; NMR data: see Table 1; HR-ESIMS (positive): *m/z* 289.1418 ([M+Na]^+^, C_15_H_22_O_4_Na; calcd. 289.1416), 249.1490 [M+H-H_2_O]^+^, 231.1385 [M+H-3×H_2_O]^+^, 213.1280 [M+H-3×H_2_O]^+^; HR-ESIMS (negative): *m/z* 265.1443 ([M-H]^−^, C_15_H_21_O_4_; calcd. 265.1445).

*Asperaculane B* (**2**). Yellowish oily liquid; ${\left[\text{α}\right]}_{D}^{\text{12}}$ = +94.5 (c 0.24, MeOH); UVλ_max_ = 236 nm; CD (MeOH) λ_max_ (Δε): 213 (+8.19), 323 (−0.63); IR (KBr) ν 3393, 2969, 2933, 1686, 1603, 1458, 1390, 1270, 1204, 1136, 1029 cm^−1^; NMR data: see Table 1; HR-ESIMS: *m/z* 237.1484 ([M+H]^+^, C_14_H_21_O_3_; calcd 237.1485), 259.1302 ([M+Na]^+^, C_14_H_20_O_3_Na; calc 259.1310), 219.1379 [M+H-H_2_O]^+^, 201.1272 [M+H-2×H_2_O]^+^, 173.1319 [M+H-2×H_2_O-CO]^+^.

*Aculene A* (**3**). Yellowish solid; ${\left[\text{α}\right]}_{D}^{\text{12}}$ = +0.72 (c 0.11, MeOH); UV λ_max_ = 241, 348 nm; CD (MeOH) λ_max_ (Δε): 242 (−12.5), 322 (+1.40), 362 (−5.28) nm; IR (KBr) ν 3427, 2976, 2944, 1744, 1685, 1409, 1238, 1203, 1130, 1029, 835, 798, 721 cm^−1^; HR-ESIMS (positive): *m/z* 316.1917 ([M+H]^+^, C_19_H_26_NO_3_; calcd 316.1907).

*Aculene B* (**4**). Yellowish solid; ${\left[\text{α}\right]}_{D}^{\text{12}}$ = +80.1 (c 0.12, MeOH); UVλ_max_ = 238 nm; CD (MeOH) λ_max_ (Δε): 216 (+9.28), 257 (−0.61), 322 (–1.20); IR (KBr) ν 3429, 2968, 2934, 1744, 1693, 1606, 1416, 1372, 1267, 1202, 1133, 1098, 864, 827, 799, 721 cm^−1^; HR-ESIMS *m/z* 318.2073 ([M+H]^+^, C_19_H_28_NO_3_; calcd 318.2064).

*Aculene C* (**5**). Yellowish oily liquid; ${\left[\text{α}\right]}_{D}^{\text{12}}$ = −26.4 (c 0.17, MeOH); UVλ_max_ = 242, 353 nm; CD (MeOH) λ_max_ (Δε): 200 (−1.85), 221 (+4.82), 242 (−1.88), 298 (+1.18), 360 (−1.05) nm; IR (KBr) ν 3400, 2972, 2937, 1687, 1581, 1454, 1379, 1275, 1205, 1138, 1067, 1026, 871, 803, 723 cm^−1^; HR-ESIMS: *m/z* 219.1387 [M+H]^+^ (C_14_H_19_O_2_; calcd 219.1385).

*Aculene D* (**6**). Yellowish oily liquid; ${\left[\text{α}\right]}_{D}^{\text{12}}$ = +112.7 (c 0.10, MeOH); UVλ_max_ = 241 nm; CD (MeOH) λ_max_ (Δε): 214 (+17.7), 265 (−0.46); 321 (−1.91); IR (KBr) v 3427, 2969, 2937, 1690, 1605, 1457, 1373, 1268, 1204, 1136, 1099, 1028, 866 cm^−1^; NMR data: see Table 1; HR-ESIMS *m/z* 221.1542 ([M+H]^+^, C_14_H_21_O_2_; calcd. 221.1542).

### 3.4. Absolute Configuration Determination

The stereochemistry of the proline residue was determined by the advanced Marfey’s method (see the Supporting Information) [22].

### 3.5. Computational Section

A preliminary conformational search was performed on the basis of a molecular mechanical method by CONFLEX6.7 with the MMFF94S force field [20]. Stable Conformers (within 3 kcal/mol) of compound **3** were saved and further optimized at B3LYP/6-31+G(d,p) level in Gaussian 09 software package [21]. Other compounds (**1**, **4**, **5** and **6**) conformers were further optimized at B3LYP/6-31+G(d,p) level. Frequency was calculated at the same level of theory. The stable conformers with populations greater than 1% and without imaginary frequencies were submitted to ECD calculation by the TDDFT (CAM-B3LYP) method associated with CPCM solvent model in methanol. Compound **3** was calculated at SVP level, and the compounds **1**, **4**, **5** and **6** were calculated at TZVP level. The excitation energies (E), oscillator strength (f), rotatory strength in velocity form (Rvel), and rotatory strength in length form (Rlen) of the lowest 30 excited states were calculated. ECD spectra of different conformers were simulated using SpecDis [27] with a half-bandwidth of 0.3 eV. The final ECD spectra were generated according to the Boltzmann-calculated distribution of each conformer.

### 3.6. Cytotoxicity Assay

*In vitro* cytotoxicity of these isolated compounds was assessed by the sulforhodamine B (SRB) assay as described previously by our group [26].

## 4. Conclusions

In conclusion, six sesquiterpenoids, including a new *ent*-daucane sesquiterpenoid, asperaculane A (**1**) and five nordaucanes, a new asperaculane B (**2**) and four aculenes A–D (compounds **3**–**6**), have been identified from the fungus *A. aculeatus*. The absolute configurations of **1**–**6** were determined for the first time by ECD. The present study revealed the asperaculane or aculene family is the antipode of most daucanes isolated from plant species. Asperaculane A (**1**) is the first example of an *ent*-daucane-type sesquiterpene found in natural products.

## Supplementary Materials

2D-NMR and UV spectra for compounds **1**–**2** and aculene D (6) as well as the stereochemistry of the proline residue can be accessed at: http://www.mdpi.com/1420-3049/20/01/0325/s1.
