# Supplementary material for: Asperaculanes A and B, Two Sesquiterpenoids from the Fungus Aspergillus aculeatus
Source: Molecules. 2014 Dec 25;20(1):325–34. doi: 10.3390/molecules20010325 (PMC6272214; doi:10.3390/molecules20010325)
Supplement: Supplementary file 1 [file molecules-20-00325-s001.pdf]

Supplementary Materials

Table of Contents

Figure S1. ^{1}H-NMR spectrum of compound **1**. 2
Figure S2. ^{13}C-NMR spectrum of compound **1**. 2
Figure S3. HMQC spectrum of compound **1** 3
Figure S4. COSY spectrum of compound **1**. 3
Figure S5. HMBC spectrum of compound **1**. 4
Figure S6. NOESY spectrum of compound **1** (in DMSO-d_6 and CD_3OD). 5
Figure S7. ^{1}H-NMR spectrum of compound **2**. 6
Figure S8. ^{13}C-NMR spectrum of compound **2**. 6
Figure S9. HMQC spectrum of compound **2** 7
Figure S10. COSY spectrum of compound **2**. 7
Figure S11. HMBC spectrum of compound **2**. 8
Figure S12. NOESY spectrum of compound **2**. 8
Figure S13. ^{1}H-NMR spectrum of compound **6**. 9
Figure S14 ^{13}C-NMR spectrum of compound **6**. 9
Figure S15. HMQC spectrum of compound **6** 10
Figure S16. COSY spectrum of compound **6**. 10
Figure S17. HMBC spectrum of compound **6**. 11
Figure S18. NOESY spectrum of compound **6** 11
Marfey's Analysis of Compounds 3 and 4 12
Figure S19. HPLC profile of Marfey's Analysis of Compounds **3** and **4**. 12

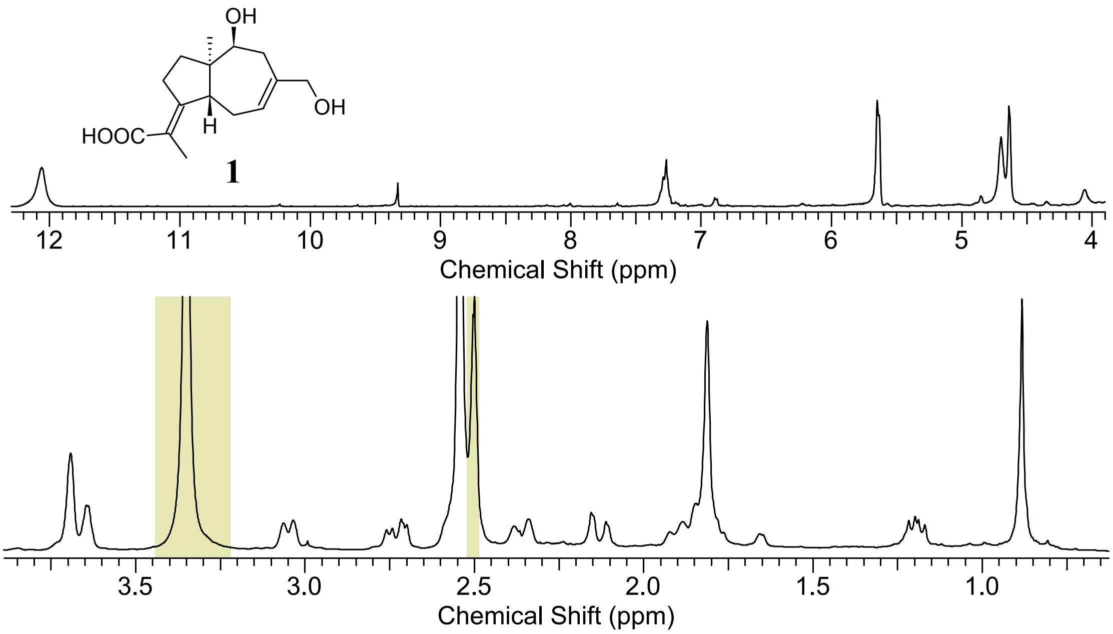

Figure S1. ^{1}H-NMR spectrum of compound **1**.

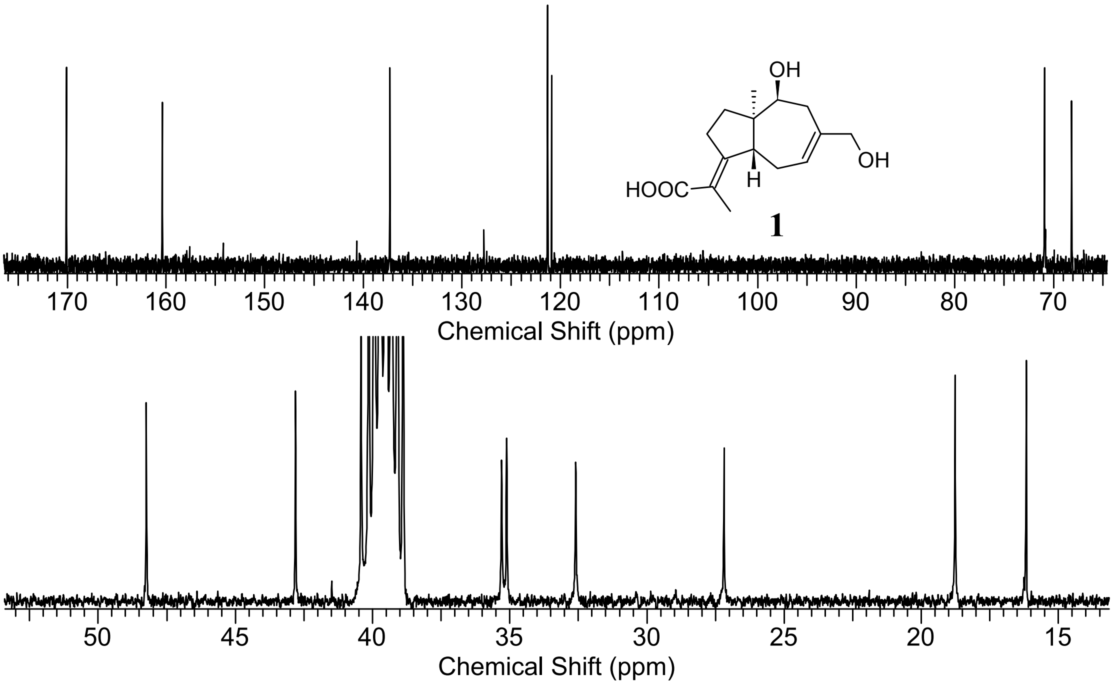

Figure S2. ^{13}C-NMR spectrum of compound **1**.

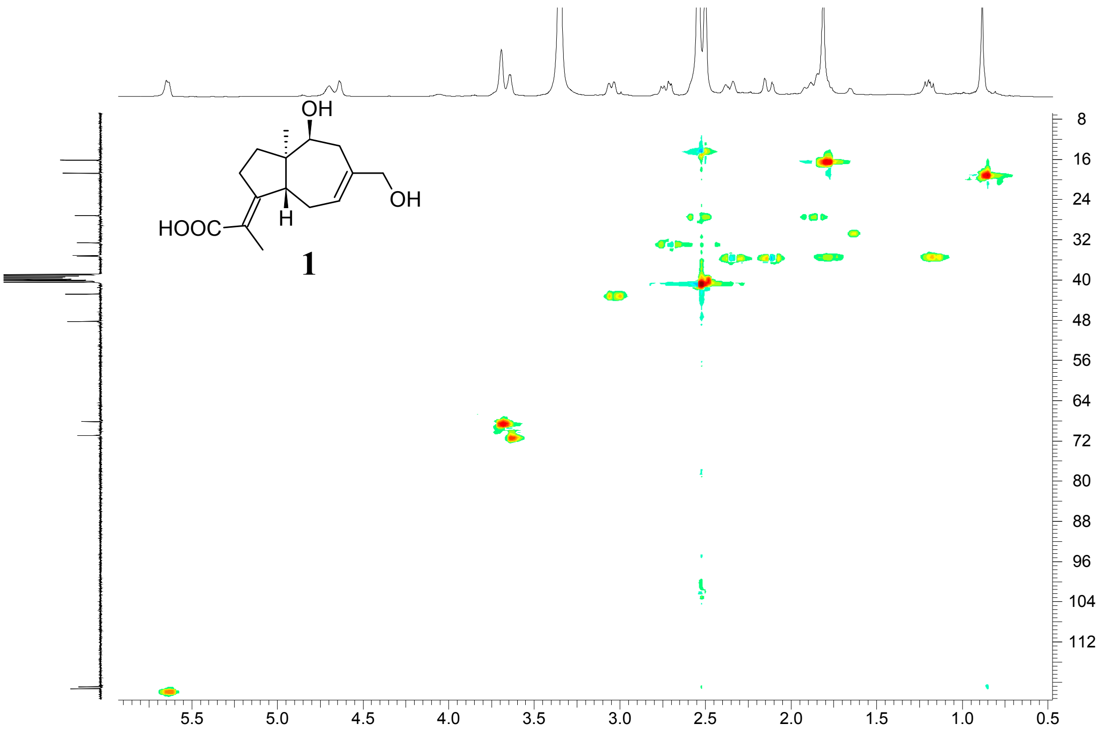

Figure S3. HMQC spectrum of compound **1**.

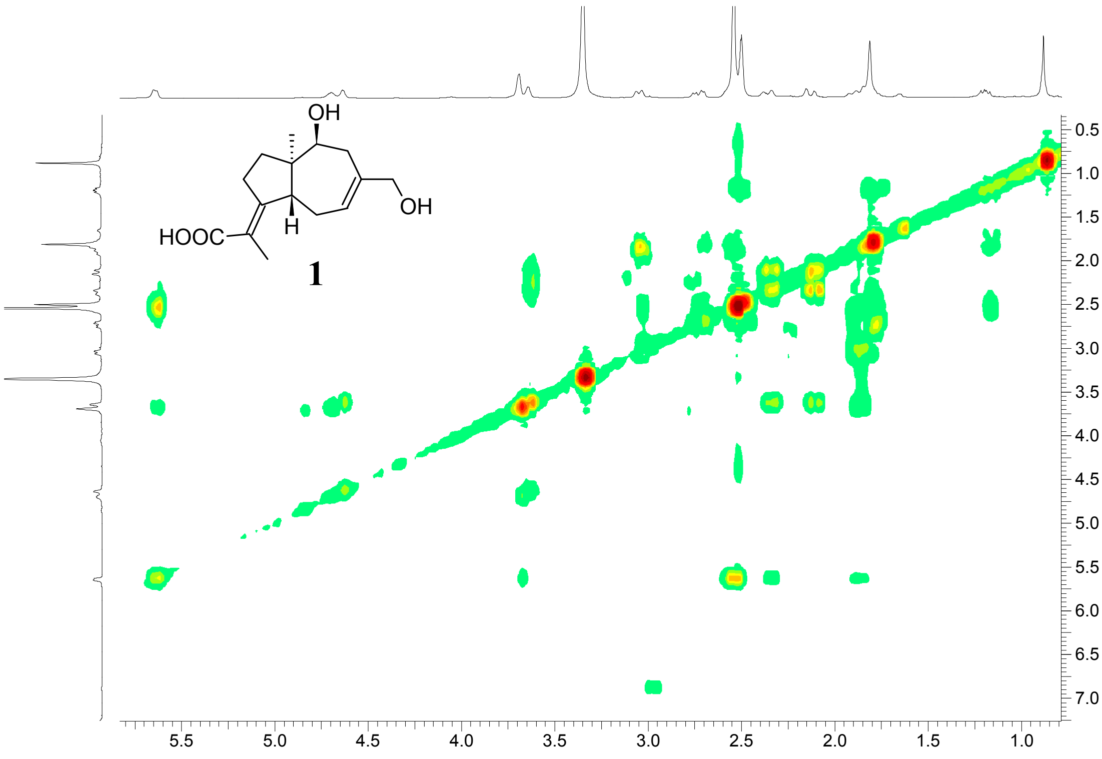

Figure S4. COSY spectrum of compound **1**.

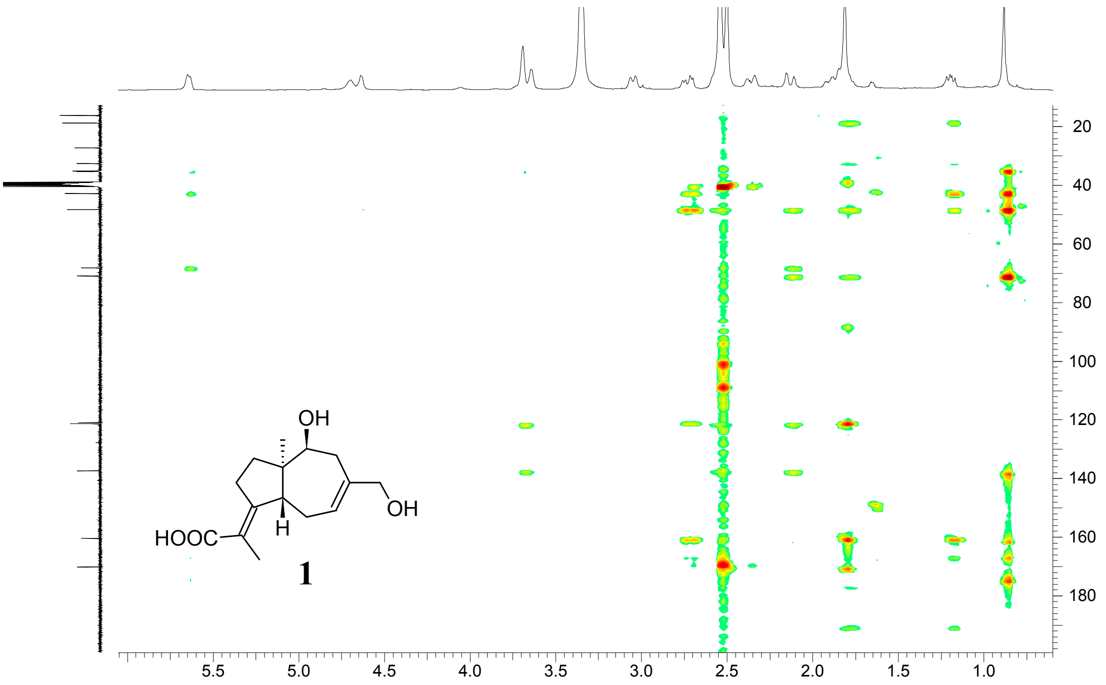

Figure S5. HMBC spectrum of compound **1**.

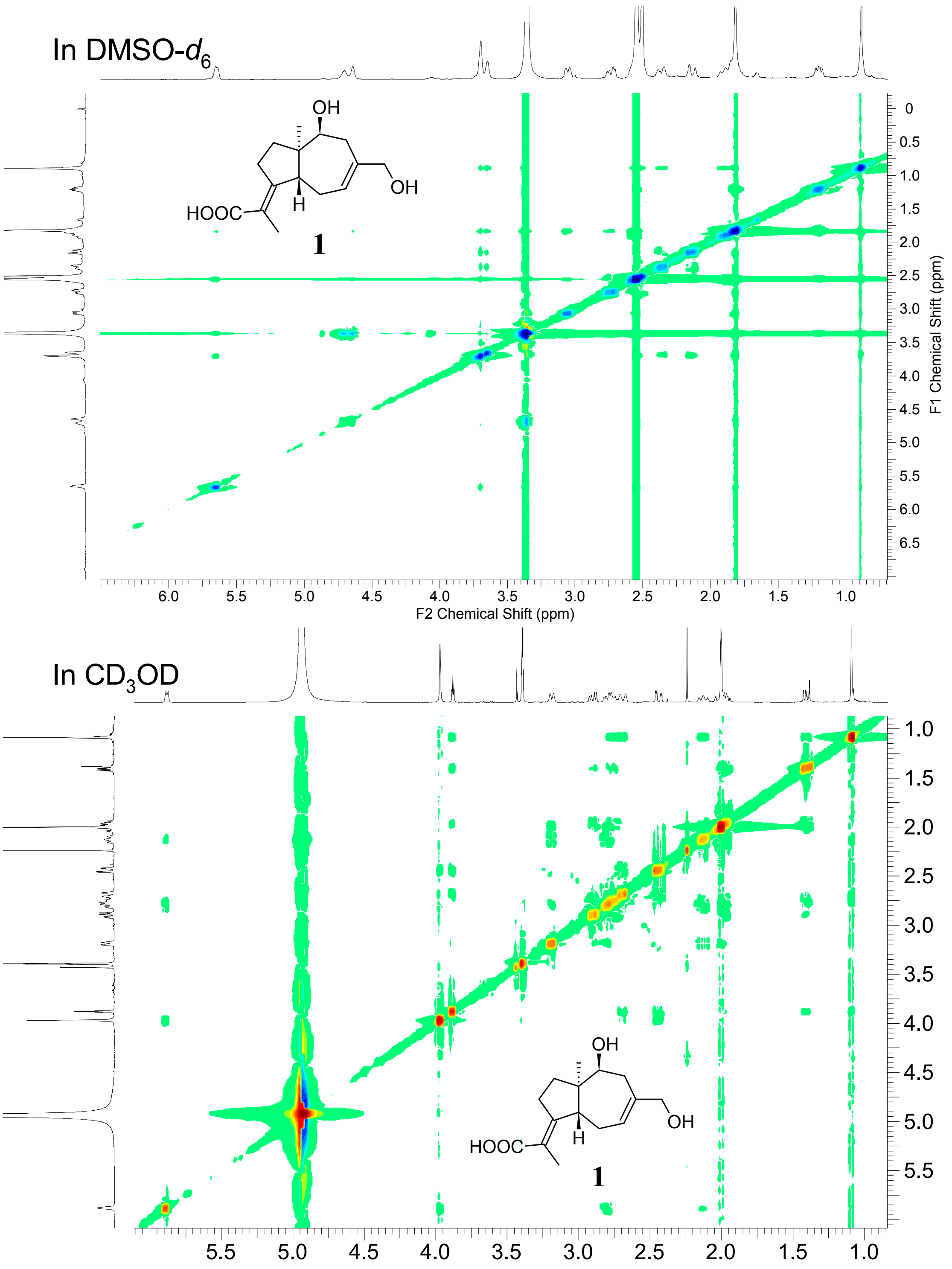

Figure S6. NOESY spectrum of compound **1** (in DMSO-*d*6 and CD3OD).

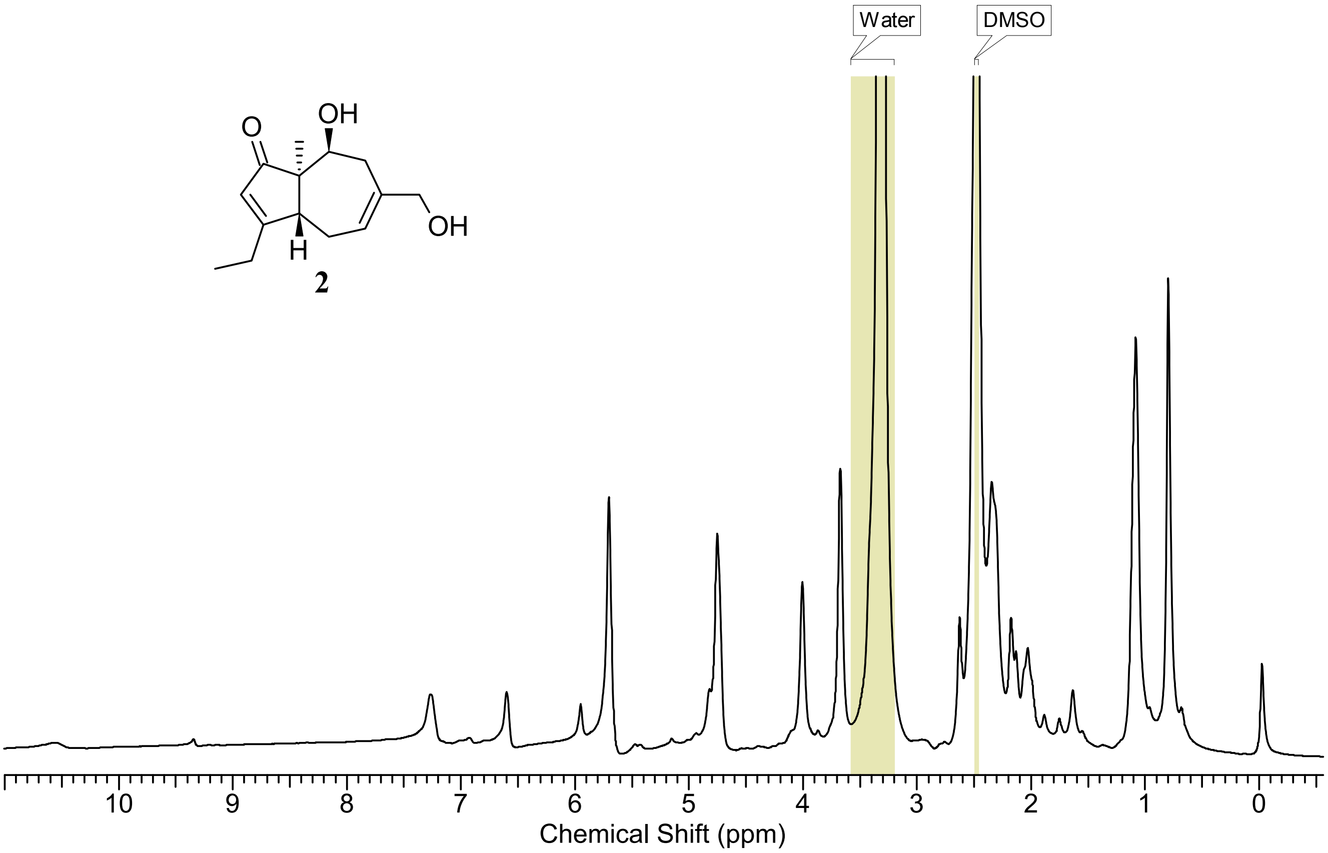

Figure S7. ^{1}H-NMR spectrum of compound **2**.

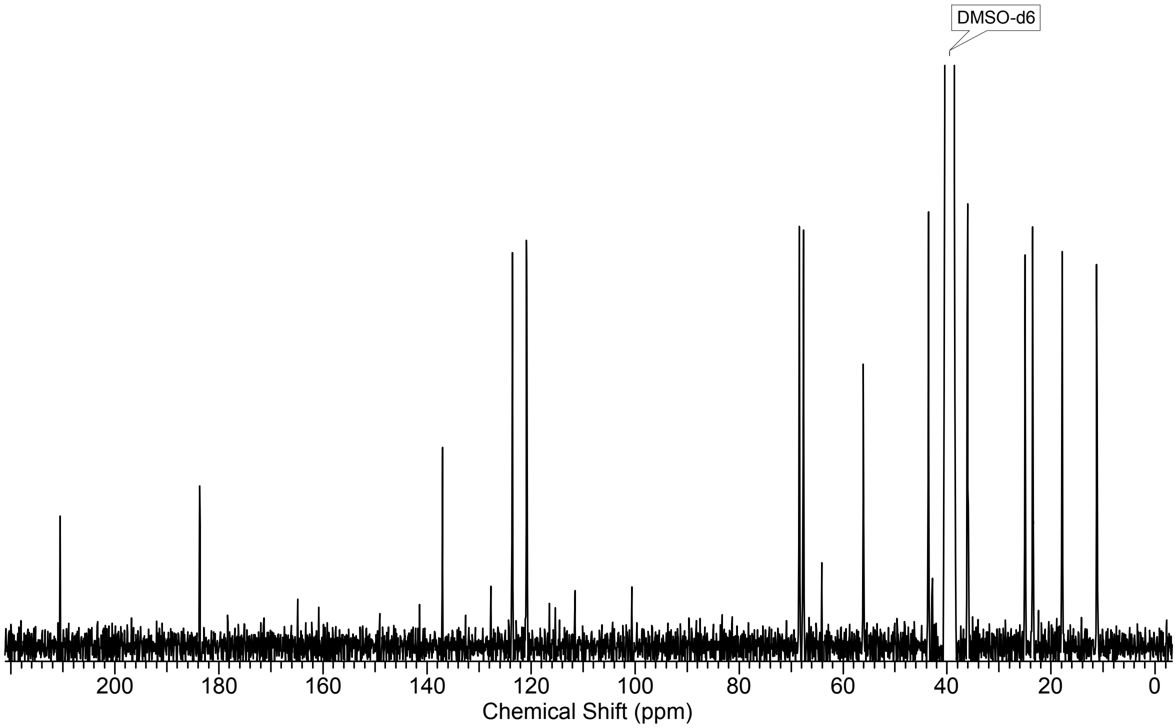

Figure S8. ^{13}C-NMR spectrum of compound **2**.

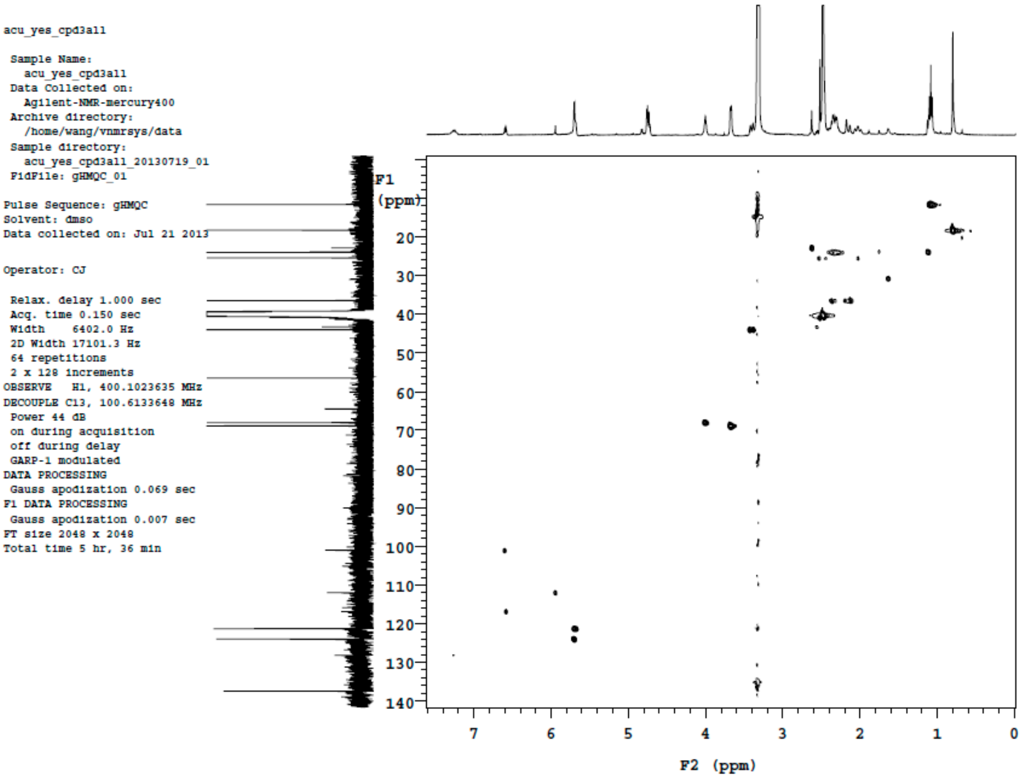

Figure S9. HMQC spectrum of compound **2**.

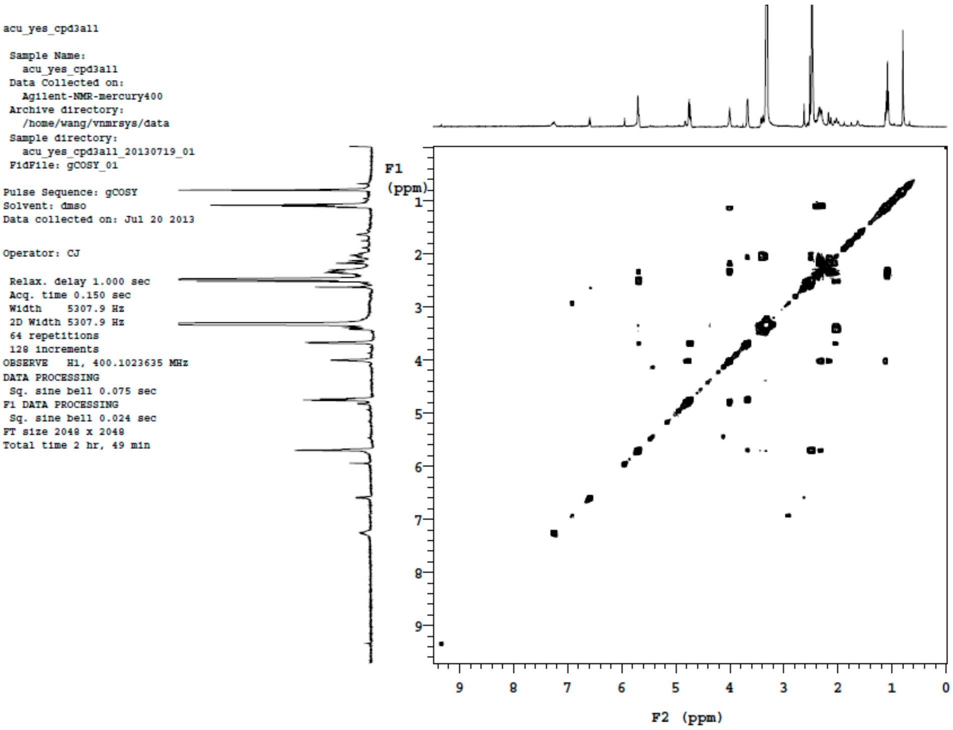

Figure S10. COSY spectrum of compound **2**.

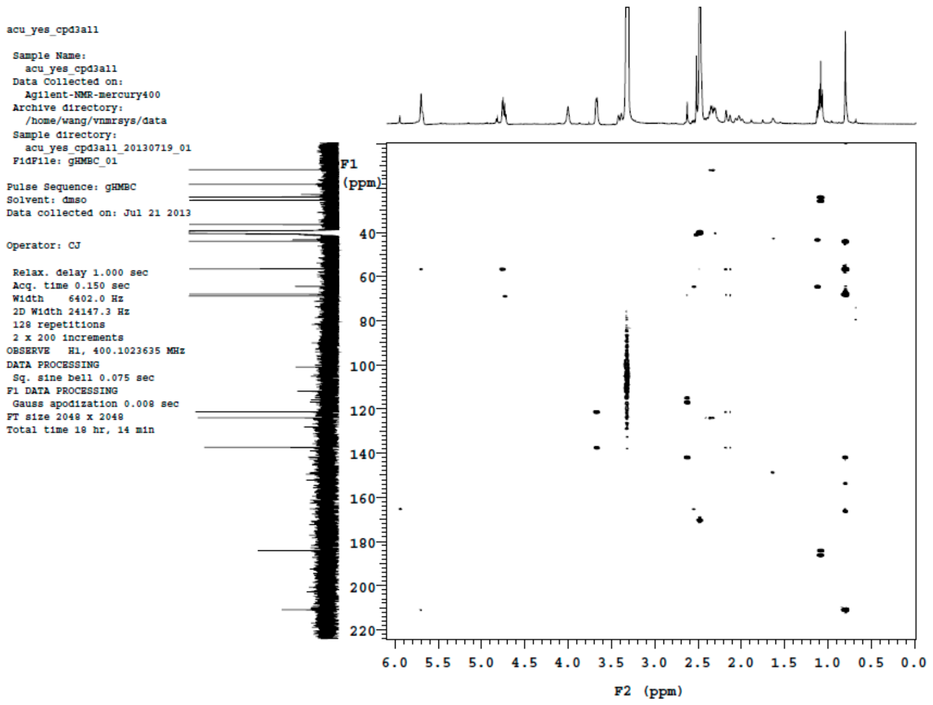

Figure S11. HMBC spectrum of compound **2**.

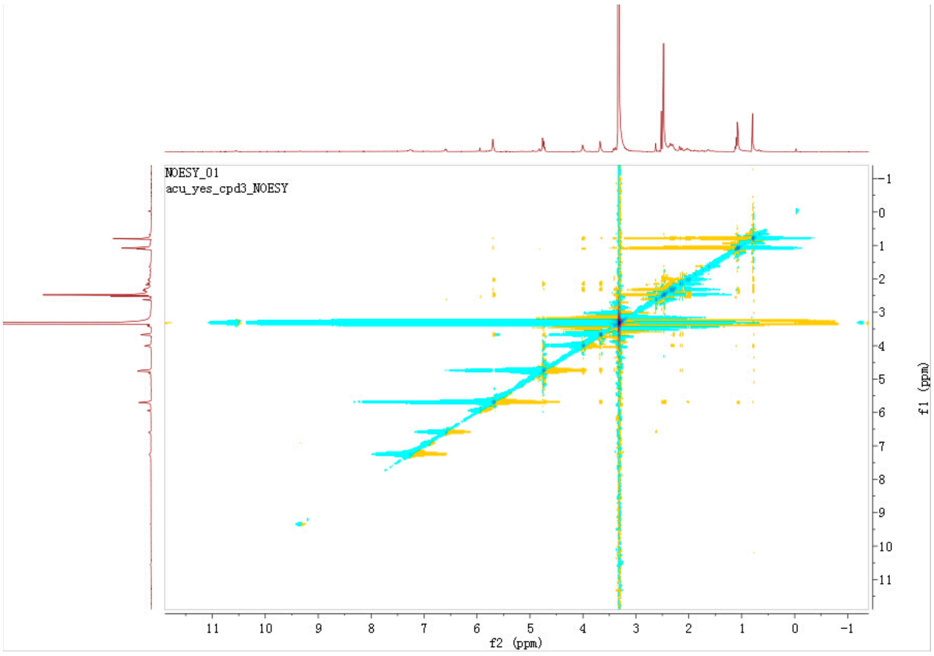

Figure S12. NOESY spectrum of compound **2**.

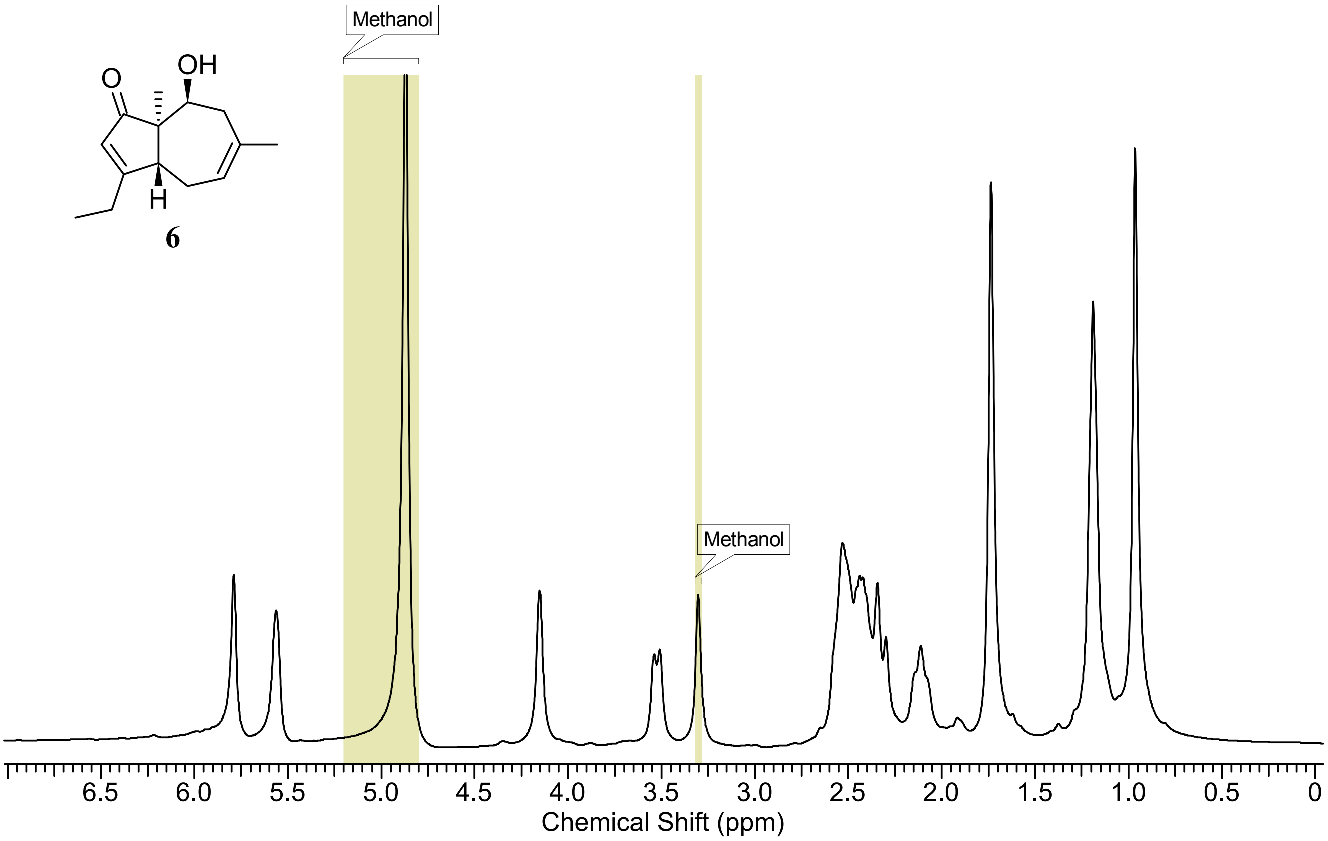

Figure S13. ^{1}H-NMR spectrum of compound **6**.

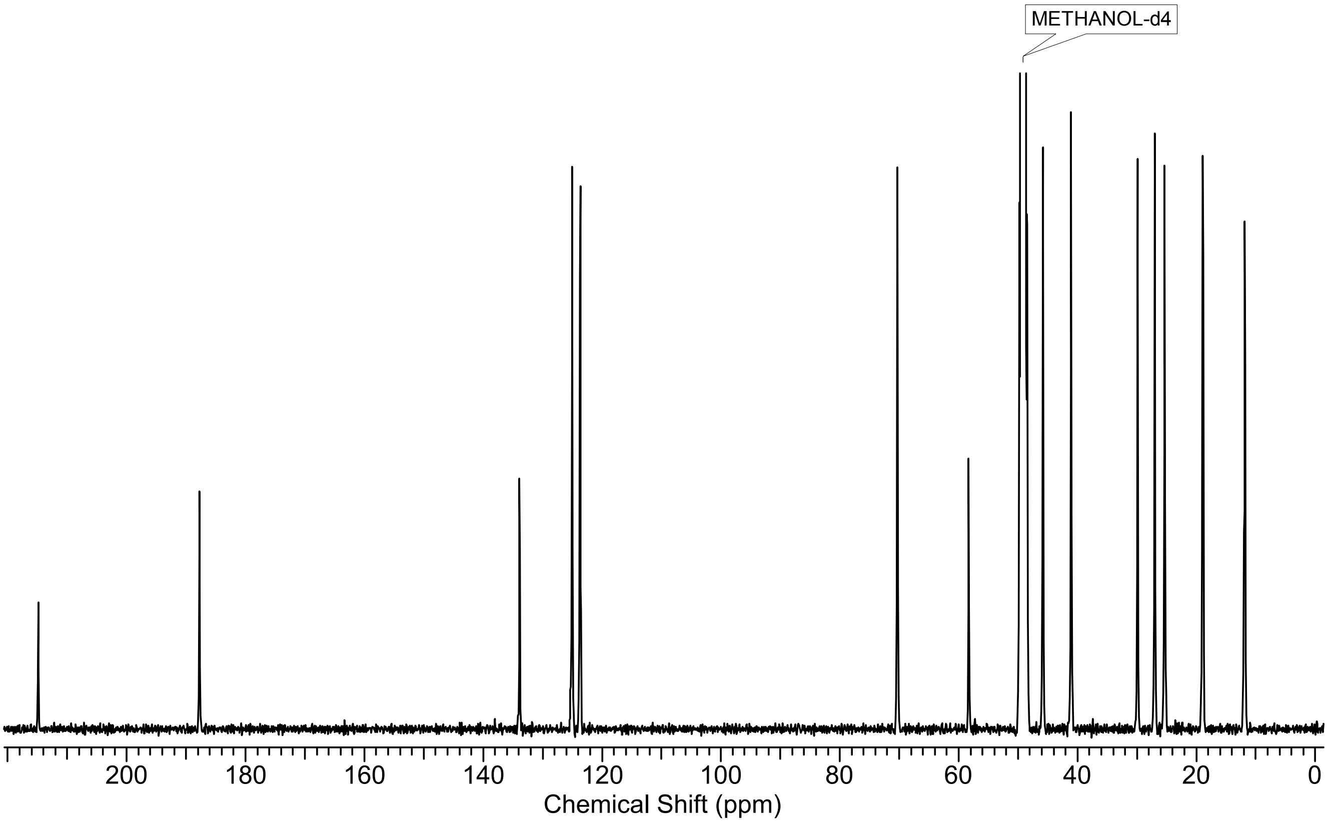

Figure S14. ^{13}C-NMR spectrum of compound **6**.

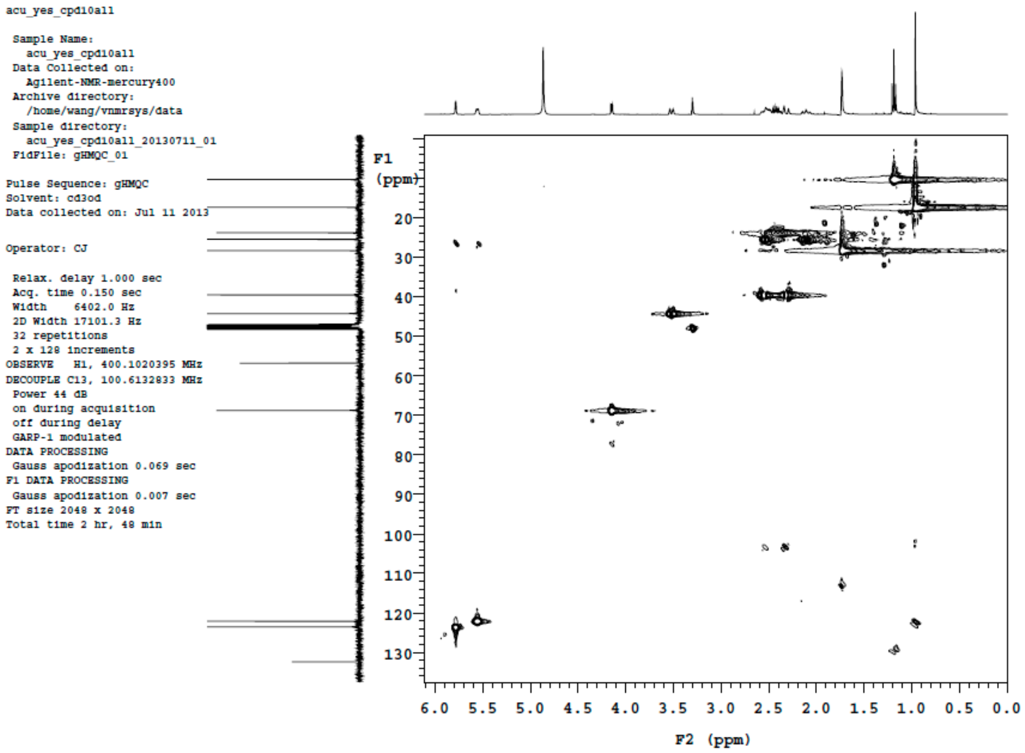

Figure S15. HMQC spectrum of compound **6**.

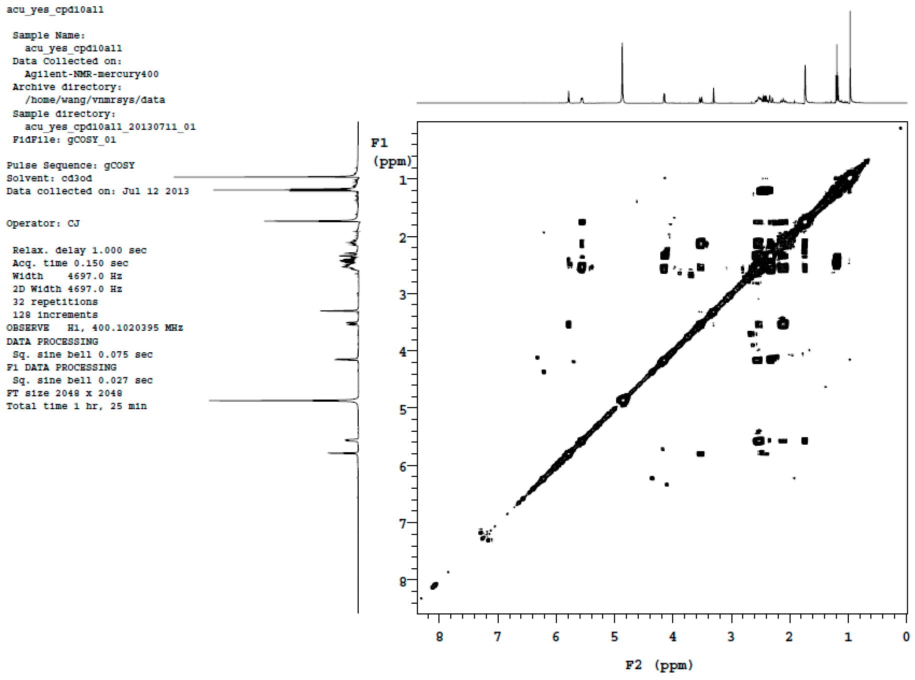

Figure S16. COSY spectrum of compound **6**.

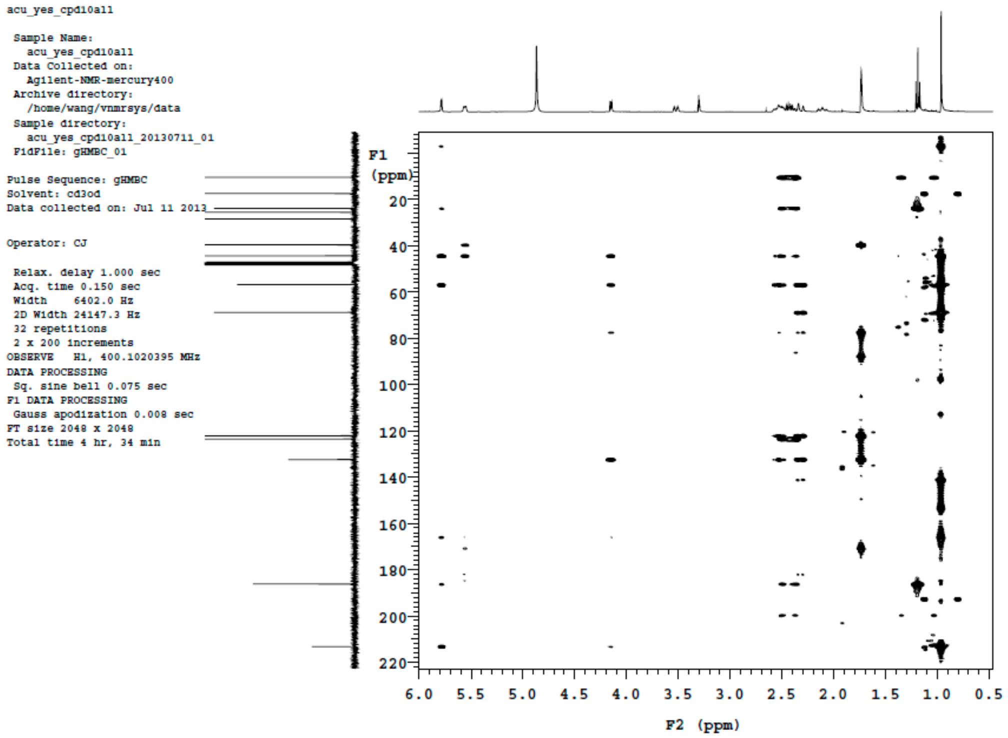

Figure S17. HMBC spectrum of compound **6**.

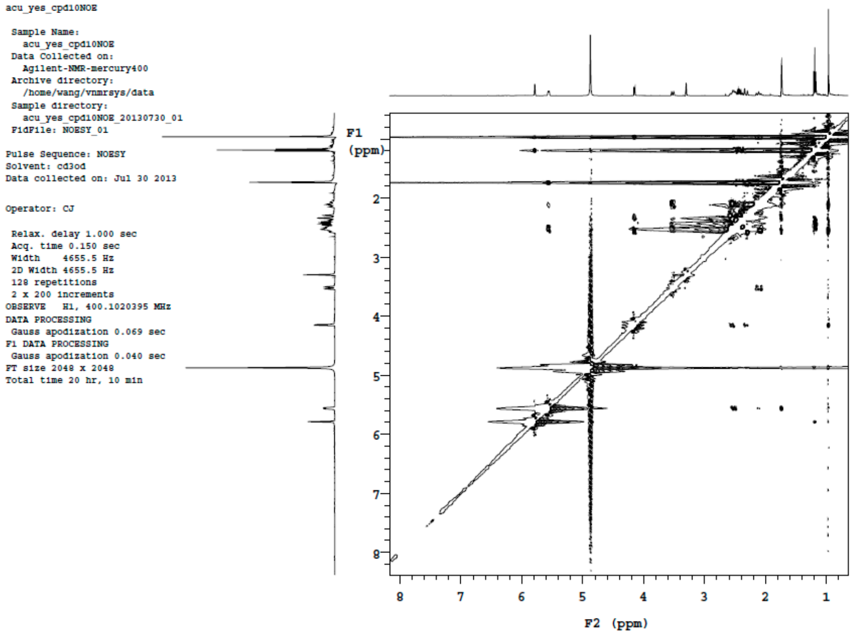

Figure S18. NOESY spectrum of compound **6**.

Marfey's Analysis of Compounds 3 and 4

The stereochemistry of the proline residue was determined by the advanced Marfey's method.

A solution of **2** or **3** (1.5 mg) in 6 N HCl (1 mL) was heated to 105 °C for 19 h. The solution was then evaporated to dryness and the residue redissolved in H_2O (250 µL). A 50 µL portion of the acid hydrolysate solution was then placed in a 1 mL reaction vial and treated with a 1% solution of 1-fluoro-2,4-dinitrophenyl-5-L-alaninamide (L-FDAA) (200 µL) in acetone followed by 1.0 M $NaHCO_3$ (40 µL). The reaction mixture was heated at 45 °C for 1 h, cooled to room temperature and then acidified with 2.0 M HCl (20 µL). In a similar fashion, the standard D- and L-Pro were derivatized with FDAA separately. The FDAA derivatives of the hydrolysates and standard amino acids were subjected to RP-HPLC analysis (Waters C18 column; 5 µm, 4.6 mm × 250 mm; 1.0 mL/min) at 30 °C using the following gradient program: solvent A, H_2O + 0.1% TFA; solvent B, MeCN; linear gradient: 0 min, 25% B, 40 min, 60% B, 30 min, 100% B; UV detection at 340 nm. The retention times for the FDAA derivatives of hydrolysates of **2** or **3** were 14.5 min; standard L-Pro, D-Pro, and were 14.5 and 19.7 min (Figure S19), respectively.

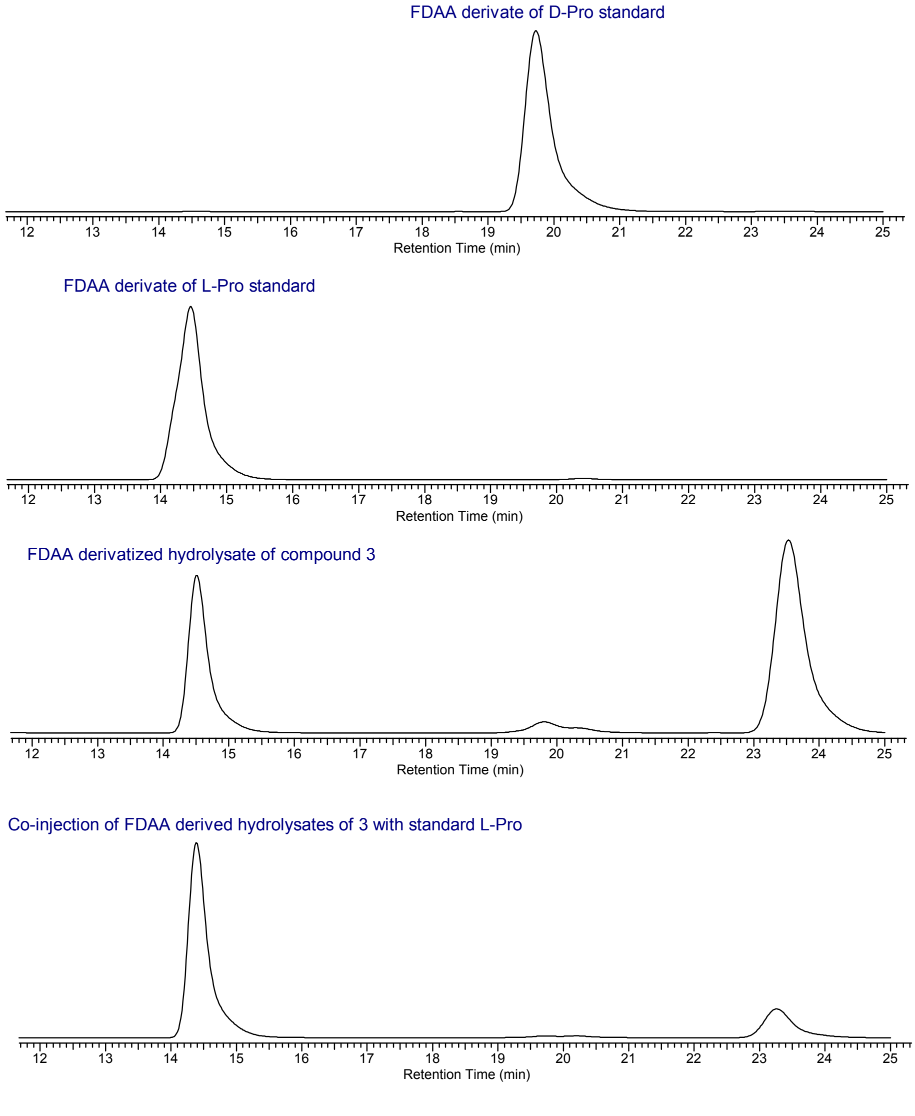

Figure S19. HPLC profile of Marfey's Analysis of Compounds **3** and **4**.
